# Dkk1 inhibition restores mandibular growth in an achondroplasia mouse model

**DOI:** 10.1242/bio.062540

**Published:** 2026-04-09

**Authors:** Briantana Raveendranathan, Valentin Estibals, Eric Pulido, Clara Lemoine, Nabil Kaci, Rachel Pereur, Mohammed Zarhrate, Sébastien Dupichaud, Emilie Dambroise, Laurence Legeai-Mallet, Martin Biosse Duplan

**Affiliations:** ^1^ Institut Imagine, UMR INSERM 1163, Université Paris Cité, 75015 Paris, France; ^2^ Genomics Platform, Institut Imagine, UMR INSERM 1163, Université Paris Cité, 75015 Paris, France; ^3^ Imaging Platform, Institut Imagine, UMR INSERM 1163, Université Paris Cité, 75015 Paris, France; ^4^ UFR Odontologie, Université Paris Cité and Hopital Bretonneau, APHP, 75018 Paris, France

**Keywords:** Craniofacial abnormalities, Mandibular condyle, Cartilage, FGFR3, Wnt signaling pathway, Chondrocyte

## Abstract

Meckel's and condylar cartilages are key to mandible development, with Meckel's cartilage acting as a template and condylar cartilage as a growth center. In achondroplasia, the most common form of genetic dwarfism, abnormalities of these cartilages lead to micrognathia, with significant functional repercussions for affected individuals. How FGFR3 overactivation in achondroplasia disrupts Meckel's and condylar cartilages is largely unknown. Our aim was to identify the pathways driving these disruptions by analyzing the genes expressed in these cartilages in a mouse model mimicking achondroplasia. Using cartilage laser-microdissection and RNA-sequencing analyses, we first compared the transcriptome of Meckel's and condylar cartilages from E16.5 embryos of control and *Fgfr3^Y367C/+^* mice. Over 900 genes were differentially expressed, including the *Dkk1* gene, which encodes an inhibitor of β-catenin-dependent Wnt signaling and was significantly overexpressed in chondrocytes of *Fgfr3* mutants in both Meckel's and condylar cartilages. Immunostaining of sections of the cartilages confirmed the high expression at the protein level. Primary cultures of Meckel's cartilage chondrocytes showed that, in *Fgfr3^Y367C/+^* mutants, activation of the canonical Wnt pathway with Wnt3a was reduced, while a Dkk1 antagonist increased Wnt activity, suggesting that Dkk1 overexpression was responsible for decreased canonical Wnt activity in mutants. In a mandible organ culture model, inhibition of Dkk1 also significantly increased the mandible size, due to an increased elongation of the condylar cartilage of mutants, as seen after tissue clearing and Sox9 immunolabeling. In this cartilage, increased proliferation and defective differentiation into hypertrophic chondrocytes was partially corrected by Dkk1 inhibition. Our data suggest that dysregulation of Wnt/β-catenin activity due to *Fgfr3* gain-of function mutation constitutes an important underlying mechanism in craniofacial defects observed in achondroplasia.

## Teaser

**Summary:** Study of an achondroplasia mouse model suggests that craniofacial defects are linked to abnormal levels of Dkk1 and Wnt/β-catenin activity in mandibular cartilages.

## INTRODUCTION

The growth and development of the mandible is controlled by several cartilages (Parada and Chai, 2015). Meckel's cartilage (MC) acts as a template for intramembranous ossification of the mandibular body during embryonic growth (Svandova et al., 2020) and plays a critical role in mandibular osteogenesis mostly through the release of mediators by chondrocytes to the surrounding osteoblasts (Shen et al., 2025). The direct contribution of MC chondrocyte to the ossification of the mandible is limited (Svandova et al., 2020). The condylar cartilage (CC), another important cartilage for the mandible's proper size and shape, is present throughout postnatal growth and directly contributes through endochondral ossification to the formation of the condylar neck and mandibular ramus (Hinton et al., 2017; Sakagami et al., 2017). The importance of these cartilages in mandibular development is illustrated by genetic diseases in which mutations in key chondrocyte genes, such as *SOX9*, *COL2A1*, or *RUNX2*, are responsible for mandibular growth defects (Chen et al., 2019). This is also the case in achondroplasia (ACH), where cartilage defects result in mandibular hypoplasia and shape anomalies with both aesthetic and functional impairments (Biosse Duplan et al., 2016; Morice et al., 2023). ACH is the leading genetic cause of disproportionate short stature, affecting one in every 25,000 births and is characterized by a rhizomelic dwarfism with short limbs (Horton et al., 2007). ACH is caused by an activating mutation of the fibroblast growth factor receptor 3 (*FGFR3*) gene, leading to overactivation of the receptor and downstream pathways such as MAPK/ERK, PI3K/AKT, STAT and RAS/MAPK pathways that affect chondrocyte proliferation, differentiation and survival (Ornitz and Marie, 2015). An additional pathway that is involved in cartilage and bone development and that may be affected by FGFR3 overactivation is the Wnt signaling pathway (Shung et al., 2012; Sun et al., 2020). Fine regulation of the Wnt/β-catenin pathway controls osteochondro-progenitor differentiation, transdifferentiation of chondrocytes into osteoblast precursors and initiation of chondrocyte hypertrophy (Oichi et al., 2020).

The management of ACH has evolved enormously in recent years, with the emergence of pharmacological treatments that correct the molecular effects of FGFR3 overactivation (Legeai-Mallet and Savarirayan, 2020) and which may serve as alternatives to repeated surgical interventions in the future.

The *Fgfr3^Y367C/+^* mouse model, which carries a single mutated allele of *Fgfr3*, faithfully recapitulates the pathophysiology and numerous clinical manifestations of ACH (Pannier et al., 2009) and, for this reason, has enabled the preclinical development of the different pharmacological approaches to ACH that are either approved or currently in clinical trials (Lorget et al., 2012; Komla-Ebri et al., 2016; Demuynck et al., 2024). We have shown that this model reproduces the craniofacial features of individuals with ACH, notably mandibular hypoplasia and defective elongation of the ramus (Biosse Duplan et al., 2016). These defects are caused by a disruption of mandibular cartilage (MC and CC) homeostasis, where Fgfr3 overactivation affects the proliferation and differentiation of chondrocytes, leading to smaller and fewer hypertrophic chondrocytes. Their correction through the inhibition of Fgfr3 tyrosine kinase activity improves mandibular development (Biosse Duplan et al., 2016).

Despite these advances, the identification of new pharmacological targets within the various cartilages remains a major issue: *FGFR3* mutations disrupt numerous signaling pathways, whereas current therapies target only a limited number of these, and each patient's specific context (including age, underlying mutation and severity of the clinical manifestations) may necessitate individualized targeting. Accordingly, our objective in this study was to systematically identify, using an unbiased approach, the signaling pathways that disturb cartilage homeostasis and are responsible for the mandibular growth defects in ACH. Following microdissection and RNA-sequencing (RNA-seq) analyses of embryonic mandibular cartilages, we detected the overexpression of Dickkopf Wnt signaling pathway inhibitor 1 (Dkk1), an inhibitor of β-catenin-dependent Wnt signaling in *Fgfr3^Y367C/+^* chondrocytes. An *ex vivo* model of mandible cultures showed that Dkk1 inhibition improved mandibular growth when Fgfr3 was overactivated. Our data suggest that dysregulation of Wnt/β-catenin activity by *FGFR3* gain-of function mutation constitutes an important underlying mechanism in cranio-facial defects in ACH.

## RESULTS

### Comparison of the transcriptomes of mandibular cartilage from *Fgfr3* mutants and controls

**Fig. 1.**
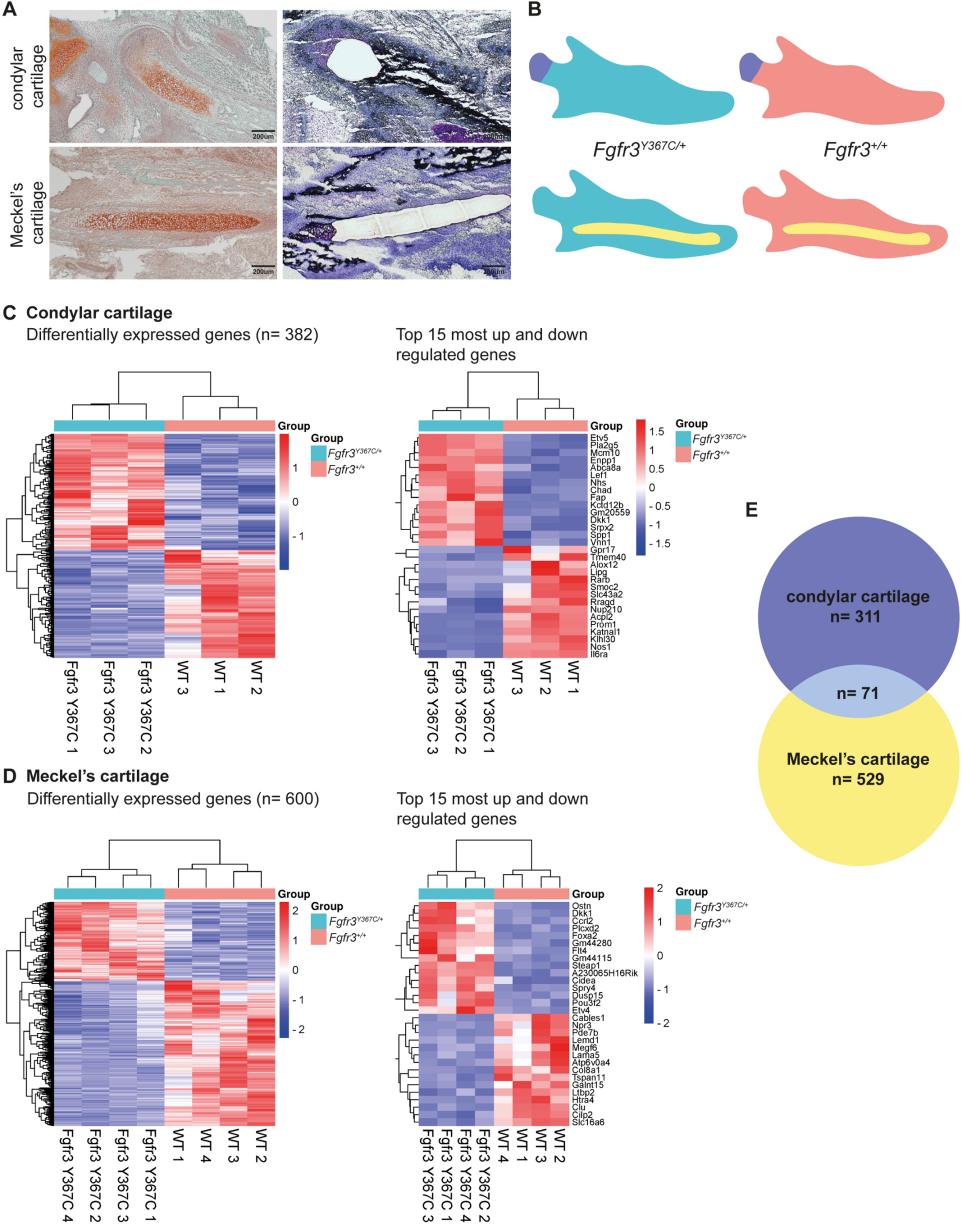
**Transcriptome analysis with RNA-seq following laser micro-dissection of the mandibular cartilages.** (A) Histologic sections of the condylar (upper row) and Meckel's (lower row) cartilages stained with Safranin-O to highlight cartilages or with Toluidine Blue following laser microdissection of the cartilages (scale bars indicated). (B) Schematic representation of the two cartilages and two genotypes analyzed. (C) Heatmap of hierarchical unsupervised clustering analysis of the six condylar cartilages analyzed (*Fgfr3^+/+^ n*=3, *Fgfr3^Y367C/+^ n*=3) and top 15 most upregulated or downregulated genes in mutants. (D) Heatmap of hierarchical unsupervised clustering analysis of the eight Meckel's cartilages analyzed (*Fgfr3^+/+^ n*=4, *Fgfr3^Y367C/+^ n*=4) and top 15 most upregulated or downregulated genes in mutants. (E) Venn diagram of the 600 differentially expressed genes (filters: *P*<0.01, mean expression ratio >2) in Meckel's cartilage and 383 in condylar cartilage (same filters) with 71 genes in common.

Hemi-mandibles of embryonic day (E)16.5 *Fgfr3^+/+^* or *Fgfr3^Y367C/+^* embryos were dissected by laser-captured microdissection (LCM), and pre-staining of the sections enabled highly accurate and reproducible isolation of the cartilage (Fig. 1A). After RNA extraction from the LCM sections, 16 samples (eight MCs and eight CCs) from eight *Fgfr3^+/+^* and eight *Fgfr3^Y367C/+^* embryos (same litters) were selected based on RNA quantity and quality and analyzed by RNA-seq. The samples from CCs most often had a low quantity of RNA (<20 ng), in relation to the size of the dissected area, and, consequently, of the eight CC samples, two samples (one *Fgfr3^+/+^* and one *Fgfr3^Y367C/+^*) were excluded from the bioinformatics analysis due to the low number of genes detected. Therefore, four MCs from *Fgfr3^+/+^* embryos were compared with four MCs from *Fgfr3^Y367C/+^* embryos, as well as three CCs from *Fgfr3^+/+^* embryos with three CCs from *Fgfr3^Y367C/+^* embryos (Fig. 1B).

**Fig. 2.**
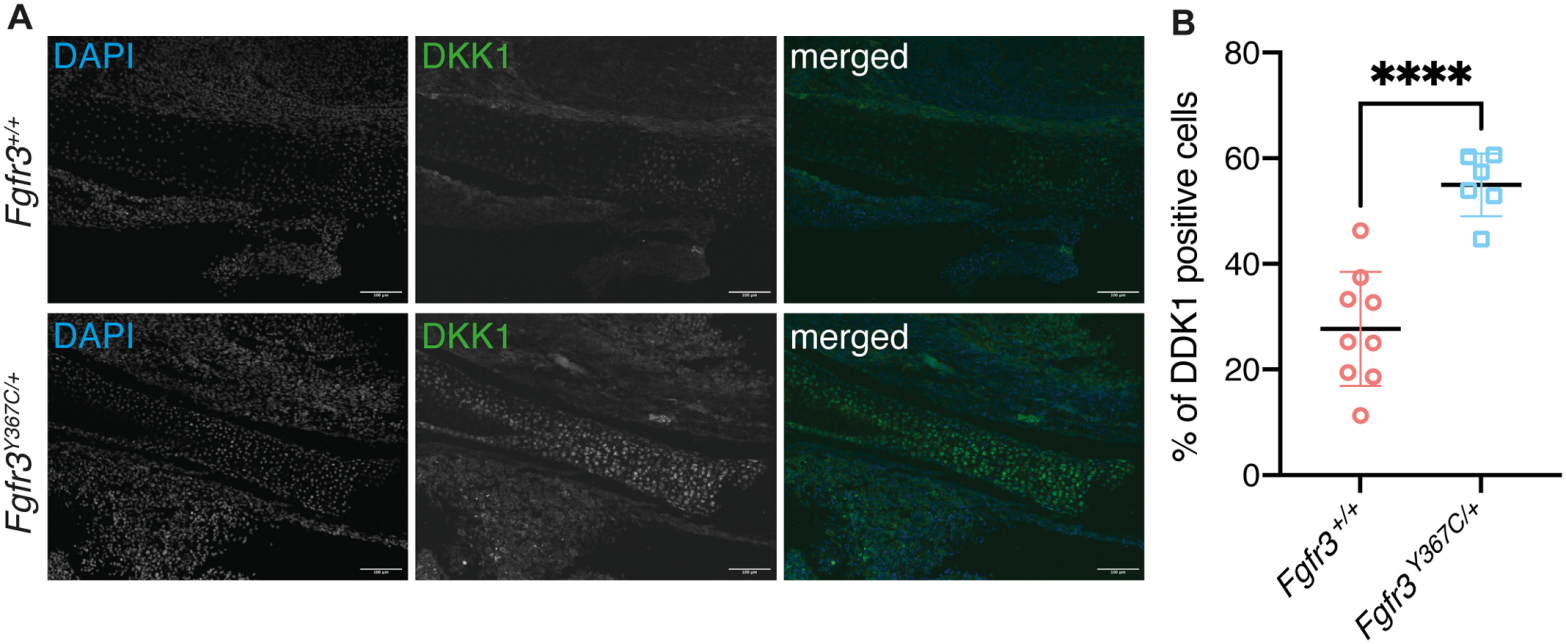
**Expression of Dkk1 in mandibular cartilages and in chondrocytes isolated from the cartilages.** (A) Visualization of all cells with nuclear staining (DAPI) and cells expressing Dkk1 in sections of Meckel's cartilage from E16.5 *Fgfr3^+/+^* and *Fgfr3^Y367C/+^* embryos. Scale bars: 100 µm. (B) Quantification of Dkk1 expression in sections of Meckel's cartilage from E16.5 embryos (*Fgfr3^+/+^n*=9, *Fgfr3^Y367C/+^ n*=6) following immunolabeling; comparison *Fgfr3^+/+^* versus *Fgfr3^Y367C/+^* (****P<0.0001; *t*-test).

Comparison between *Fgfr3* mutants and *Fgfr3^+/+^* revealed 600 differentially expressed genes (filters: *P*<0.01, mean expression ratio >2) in MC and 382 in CC (same filters) (Fig. 1C-E). Seventy-one genes were common to the two lists (Table S1), including several genes involved in Wnt and BMP signaling pathways (*Lef1*, *Spry2*, *Spry4*, *Dkk1*, *Bmp7*, *Sfrp1*). *Dkk1* was identified among the 15 most upregulated genes in *Fgfr3* mutants compared to controls, in both MC and CC (Fig. 1C,D). We confirmed that the overexpression of *Dkk1* detected by RNA-seq resulted in an increased protein abundance in mandibular cartilages from *Fgfr3* mutants (+98% compared to *Fgfr3^+/+^* embryos, *P*<0.0001; Fig. 2A,B). Because of its key role in the Wnt pathway and the relevance of the pathway in the differentiation of chondrocytes and osteoblasts, we focused on the impact of Dkk1 overexpression in the context of *Fgfr3* overactivation.

### Canonical Wnt activity in chondrocytes from *Fgfr3^Y367C/+^* mutants

We next sought to determine whether the overexpression of Dkk1 caused by the activating *Fgfr3* mutation alters Wnt activity in mandibular cartilage chondrocytes. For this, we isolated and cultured MC chondrocytes from *Fgfr3^Y367C/+^* mutant or control embryos. The majority of MC-derived cells cultured were chondrocytes as evidenced by the expression of Sox9 in more than 85% of cells (Fig. S1A). These cells also expressed Dkk1 (Fig. S1B).

**Fig. 3.**
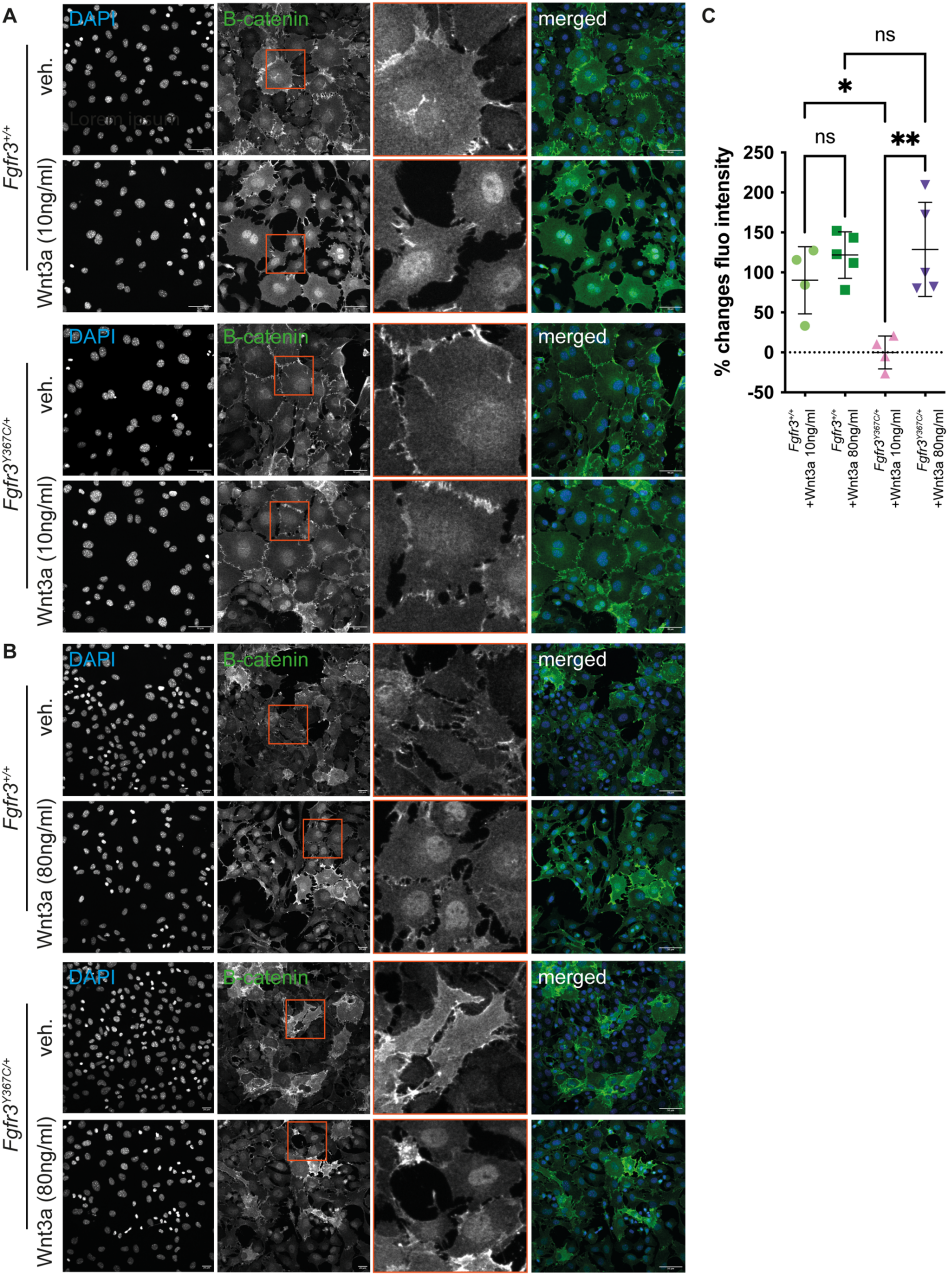
**Wnt/β-catenin signaling activity in chondrocytes from Meckel's cartilage treated with Wnt3a.** (A,B) Visualization of nuclear (DAPI) and β-catenin staining in chondrocytes from E16.5 *Fgfr3^+/+^* and *Fgfr3^Y367C/+^* embryos following stimulation with vehicle or Wnt3a at the indicated concentration. Scale bars: 50 µm. (C) Quantification of the changes in β-catenin nuclear fluorescence intensity in chondrocytes isolated from E16.5 *Fgfr3^+/+^* and *Fgfr3^Y367C/+^* embryos. Each dot represents cells isolated from one embryo [*Fgfr3^+/+^* treated with Wnt3a at 10 ng/ml compared to vehicle, *n*=4; *Fgfr3^+/+^* treated with Wnt3a at 80 ng/ml compared to vehicle, *n*=5; *Fgfr3^Y367C/+^* treated with Wnt3a at 10 ng/ml compared to vehicle, *n*=4; *Fgfr3^Y367C/+^* treated with Wnt3a at 80 ng/ml compared to vehicle, *n*=5; ns, not significant; *P<0.05, **P<0.01; one-way ANOVA followed by, when significant (*P*<0.05), the Tukey test for multiple comparison of the mean of each group].

We induced Wnt activity by treating MC chondrocytes with the ligand Wnt3a and measured the nuclear translocation of β-catenin. β-catenin accumulation was assessed by measuring the fluorescence intensity of the signal obtained by immunostaining in a mask defined by DAPI nuclear staining. β-catenin accumulation was compared between cells derived from the same embryo (*Fgfr3^+/+^* or *Fgfr3^Y367C/+^*), either stimulated with Wnt or unstimulated. We first used a low dose of Wnt3a (10 ng/ml) and observed nuclear translocation of β-catenin in *Fgfr3^+/+^* MC chondrocytes but not in *Fgfr3^Y367C/+^* cells (Fig. 3A,C). In contrast, a high dose (80 ng/ml) of Wnt3a was able to induce β-catenin accumulation in the nucleus of chondrocytes from both *Fgfr3^Y367C/+^* mutants and controls, with a similar response (Fig. 3B,C).

**Fig. 4.**
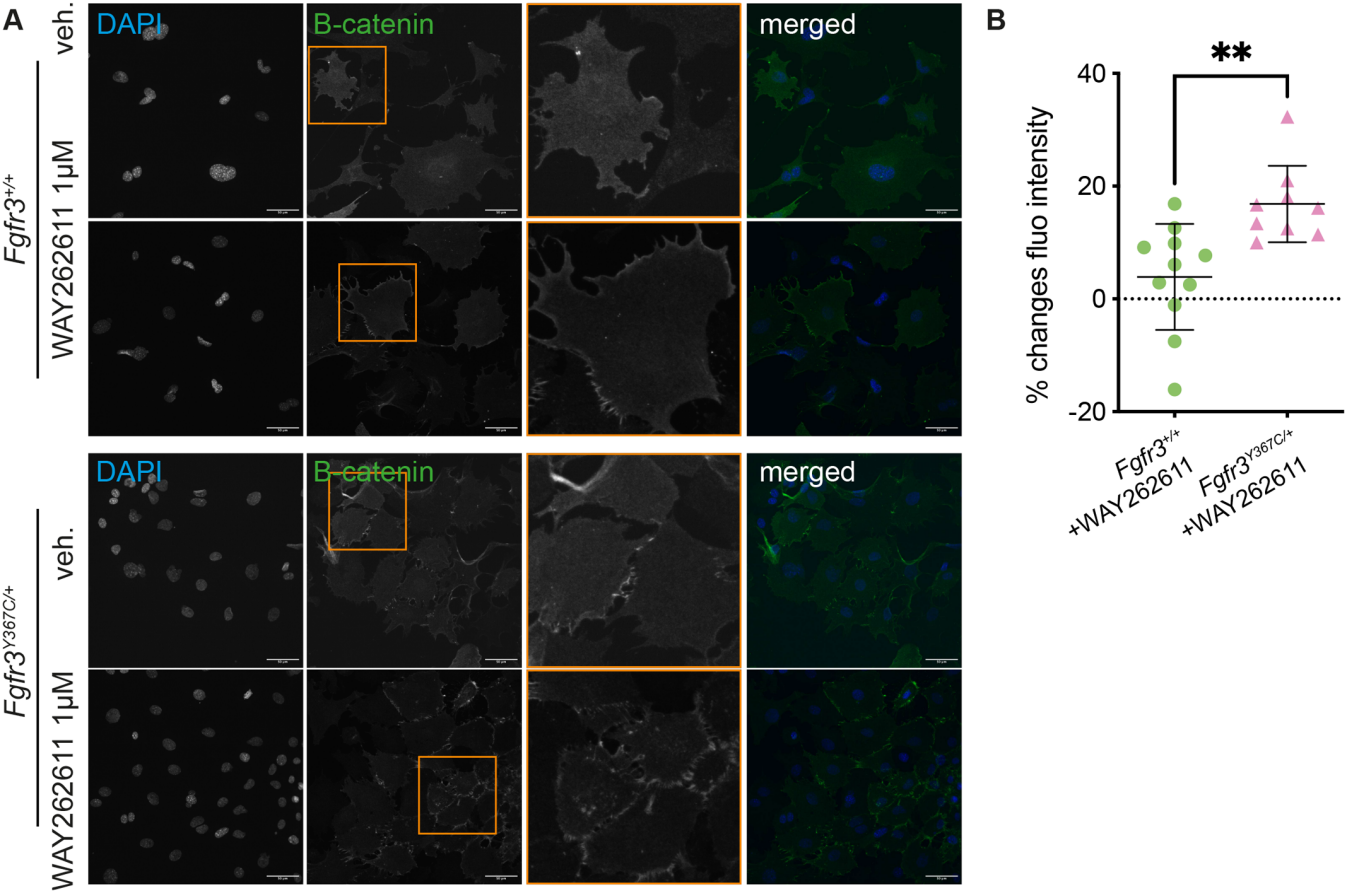
**Wnt/β-catenin signaling activity in chondrocytes from Meckel's cartilage treated with Dkk1 inhibitor.** (A) Visualization of nuclear (DAPI) and β-catenin staining in chondrocytes from E16.5 *Fgfr3^+/+^* and *Fgfr3^Y367C/+^* embryos following treatment with vehicle or the Dkk1 inhibitor WAY262611 at the indicated concentration. Scale bars: 50 µm. (B) Quantification of the changes in β-catenin nuclear fluorescence intensity in chondrocytes isolated from E16.5 *Fgfr3^+/+^* and *Fgfr3^Y367C/+^* embryos. Each dot represents cells isolated from one embryo [*Fgfr3^+/+^* treated with WAY262611 at 1 µM compared to vehicle, *n*=11; *Fgfr3^Y367C/+^* treated with Wnt3a at 10 ng/ml compared to vehicle, *n*=4; *Fgfr3^Y367C/+^* treated with WAY262611 at 1 µM compared to vehicle, *n*=9; **P<0.01; one-way ANOVA followed by, when significant (*P*<0.05), the Tukey test for multiple comparison of the mean of each group].

We then tested the effect of Dkk1 inhibition on β-catenin accumulation by treating MC chondrocytes with the Dkk1 inhibitor WAY262611 (Pelletier et al., 2009). We observed that the inhibitor had no effect on chondrocytes derived from *Fgfr3^+/+^* cartilage, whereas it increased β-catenin accumulation in *Fgfr3^Y367C/+^* cells (Fig. 4A,B).

Together, these experiments allowed us to conclude that MC chondrocytes from *Fgfr3^+/+^* controls and *Fgfr3^Y367C/+^* mutants presented with different β-catenin accumulation in the nucleus that could be a consequence of Dkk1 overexpression.

### *Ex vivo* inhibition of Dkk1 improves mandibular growth in *Fgfr3^Y367C/+^* mutants

**Fig. 5.**
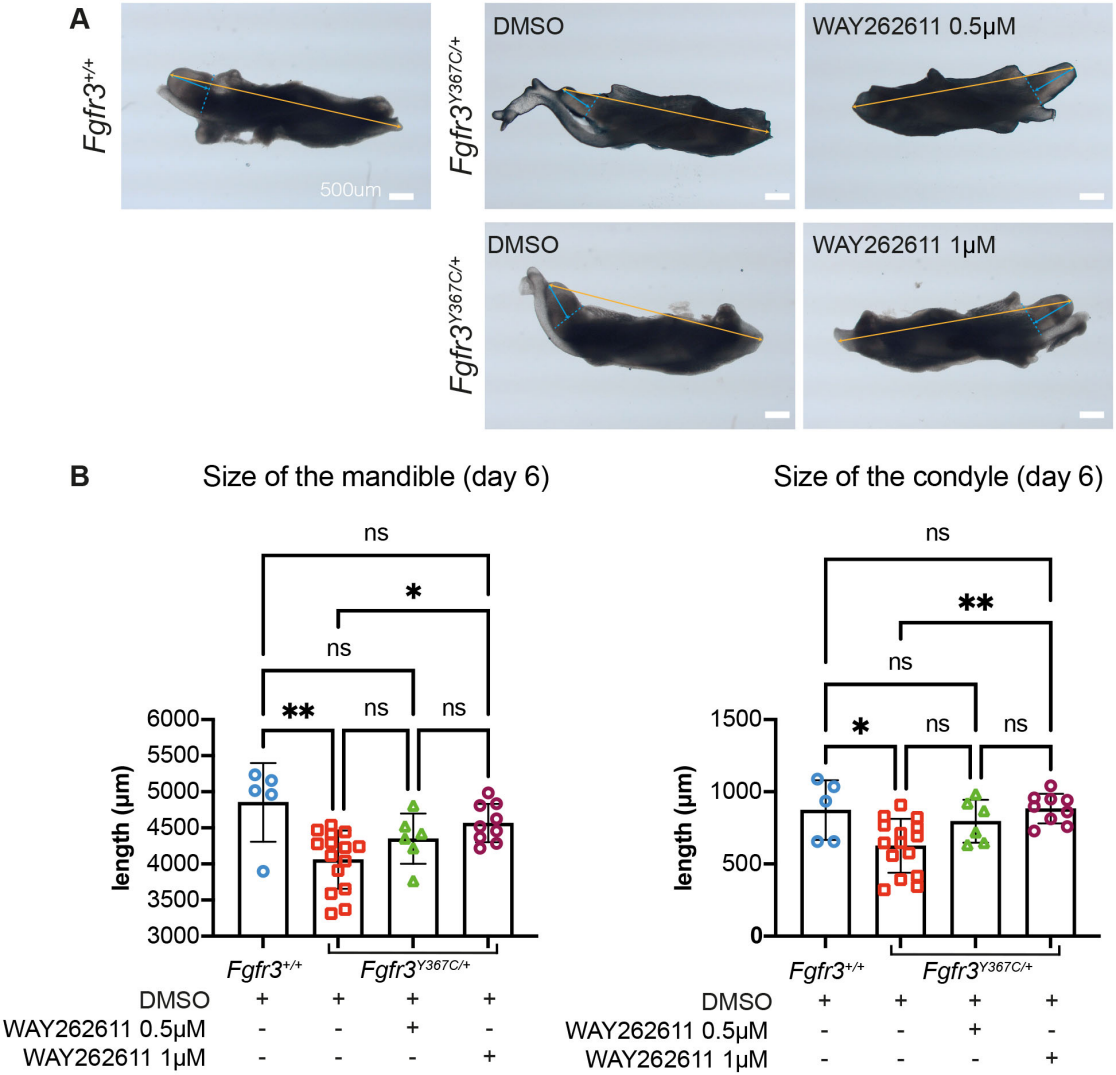
**Mandible organ cultures from *Fgfr3* mutants treated with Dkk1 inhibitor.** (A) Macroscopic view of hemi-mandibles from *Fgfr3^+/+^* and *Fgfr3^Y367C/+^* embryos cultured and treated with the Dkk1 inhibitor WAY262611 at the indicated concentration for 6 days. Blue and orange arrows indicate measurements of the condyle and total mandible. Scale bars: 500 µm. (B) Quantification of the total or condyle length of the cultured hemi-mandibles treated with vehicle or WAY262611 at the indicated concentrations. Each dot represents a hemi-mandible [*Fgfr3^+/+^* treated with vehicle *n*=5, *Fgfr3^Y367C/+^* treated with vehicle *n*=15, *Fgfr3^Y367C/+^* treated with WAY262611 0.5 µM *n*=6, *Fgfr3^Y367C/+^* treated with WAY262611 1 µM *n*=9; ns, not significant; *P<0.05, **P<0.01; one-way ANOVA followed by, when significant (*P*<0.05), the Tukey test for multiple comparison of the mean of each group].

We then investigated whether high Dkk1 expression contributes to the reduced size of the mandible and elongation of the condyle observed when Fgfr3 is overactivated. In a mandibular organ culture model (Biosse Duplan et al., 2016), we compared for each embryo the growth of one hemi-mandible treated with different concentrations of Dkk1 inhibitor or vehicle. Macroscopic analysis confirmed that growth was reduced in *Fgfr3^Y367C/+^* mutants compared with *Fgfr3^+/+^*, as reflected by the shorter mandible (−28,4%, *P*<0.01) and condyle (−16.3%, *P*<0.05) (Fig. 5A,B). Dkk1 inhibition significantly improved mandible growth in *Fgfr3^Y367C/+^* mandibles to approach the final size observed in controls (+12,5%, *P*<0.05; Fig. 5A,B). This was largely due to an increase in condylar elongation (+41,1%, *P*<0.01; Fig. 5A,B). A dose-response was observed, with a stronger effect with the 1 µM concentration than with that of 0.5 µM.

**Fig. 6.**
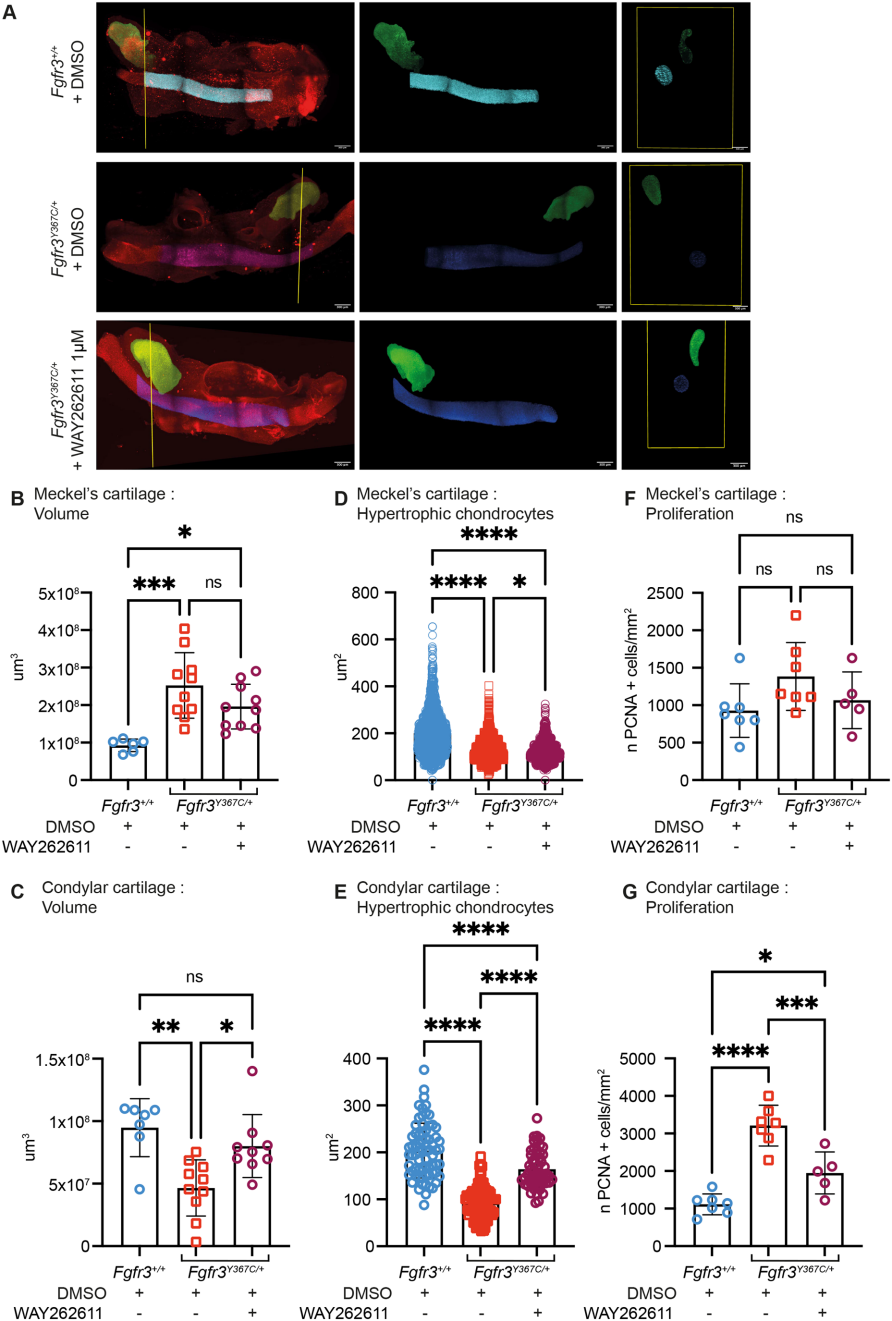
**Mandible organ cultures from *Fgfr3* mutants treated with Dkk1 inhibitor.** (A) 3D visualization of hemi-mandibles from *Fgfr3^+/+^* and *Fgfr3^Y367C/+^* embryos cultured and treated with the Dkk1 inhibitor WAY262611 at 1 µM, after tissue clearing, Sox9 immunolabeling (red channel) and imaging with a light-sheet microscope. Meckel's (blue channel) and condylar (green channel) cartilages were segmented using cross-sectional views (in yellow). Scale bars: 300 µm. (B) Quantification of Meckel's cartilage volume of cultured hemi-mandibles treated with vehicle or WAY262611 at 1 µM following segmentation. Each dot represents a hemi-mandible (*Fgfr3^+/+^* treated with vehicle *n*=6, *Fgfr3^Y367C/+^* treated with vehicle *n*=10, *Fgfr3^Y367C/+^* treated with WAY262611 1 µM *n*=10; ns, not significant; *P<0.05, ***P<0.001). (C) Quantification of condylar cartilage volume of cultured hemi-mandibles treated with vehicle or WAY262611 at 1 µM following segmentation. Each dot represents a hemi-mandible (*Fgfr3^+/+^* treated with vehicle *n*=7, *Fgfr3^Y367C/+^* treated with vehicle *n*=10, *Fgfr3^Y367C/+^* treated with WAY262611 1 µM *n*=9; ns, not significant; *P<0.05, **P<0.01). (D) Quantification of hypertrophic chondrocytes size in Meckel's cartilages of hemi-mandibles from *Fgfr3^+/+^* and *Fgfr3^Y367C/+^* embryos cultured and treated with WAY262611. Each dot represents a cell (*n*>1000 cells for each genotype and condition; *P<0.05, ****P<0.0001). (E) Quantification of hypertrophic chondrocytes size in condylar cartilages of hemi-mandibles from *Fgfr3^+/+^* and *Fgfr3^Y367C/+^* embryos cultured and treated with WAY262611. Each dot represents a cell (*n*>50 cells for each genotype and condition; ****P<0.0001). (F) Quantification of chondrocytes proliferation in Meckel's cartilages of hemi-mandibles from *Fgfr3^+/+^* and *Fgfr3^Y367C/+^* embryos cultured and treated with WAY26261. Each dot represents a hemi-mandible (*Fgfr3^+/+^* treated with vehicle *n*=7, *Fgfr3^Y367C/+^* treated with vehicle *n*=7, *Fgfr3^Y367C/+^* treated with WAY262611 *n*=5; ns, not significant). (G) Quantification of chondrocytes proliferation in condylar cartilages of hemi-mandibles from *Fgfr3^+/+^* and *Fgfr3^Y367C/+^* embryos cultured and treated with WAY26261. Each dot represents a hemi-mandible (*Fgfr3^+/+^* treated with vehicle *n*=7, *Fgfr3^Y367C/+^* treated with vehicle *n*=7, *Fgfr3^Y367C/+^* treated with WAY262611 *n*=5; *P<0.05, ***P<0.001, ****P<0.0001). One-way ANOVA followed by, when significant (*P*<0.05), the Tukey test for multiple comparison of the mean of each group.

To better assess the effect of Dkk1 inhibition on the growth of *Fgfr3^Y367C/+^*and control mandibles and measure volumetric changes of mandibular cartilages, hemi-mandibles were cleared at the end of *ex vivo* cultures. After three-dimensional imaging of the hemi-mandibles immunolabelled with Sox9, and segmentation of the different mandibular cartilages, we were able to precisely quantify the volume of each cartilage (Fig. 6A). In the absence of treatment, we observed a larger MC in the *Fgfr3^Y367C/+^* mandibles compared with *Fgfr3^+/+^* (Fig. 6B), while the condyle cartilage was less voluminous in the mutants (Fig. 6C). When Dkk1 was inhibited, there was a significant increase in the volume of the condyle cartilage in *Fgfr3^Y367C/+^* mutants, to achieve an almost total recovery compared to the control (+72%, *P*<0.05). There was a trend towards a less voluminous MC with Dkk1 inhibition (−22%, *P*=0.16)*.*

In view of the critical role of Wnt signaling in chondrocyte fate determination and the impact of enhanced Fgfr3 signaling on their proliferation and differentiation, we investigated the effect of Dkk1 inhibition on mandibular cartilage chondrocytes. As expected, differentiation was defective in mandibular cartilages of *Fgfr3^Y367C/+^* mutants compared to *Fgfr3^+/+^*, characterized by smaller hypertrophic chondrocytes, identified following Type X collagen immunostaining (−35%, *P*<0.0001 in MC, −54%, *P*<0.0001 in CC; Fig. 6D,E; Fig. S2A,B). Inhibition of Dkk1 did not improve hypertrophic chondrocyte differentiation in MC (−5.1%, *P*<0.02; Fig. 6D; Fig. S2A), whereas it partially corrected this defect in CC and improved the average size of hypertrophic chondrocytes (+77%, *P*<0.0001; Fig. 6E; Fig. S2B). Finaly, we measured the proliferation of mandibular cartilages chondrocytes, following PCNA immunostaining. We observed a non-statistically significant increase in chondrocyte of MC from *Fgfr3^Y367C/+^* mutants (+49%, *P*=0.11; Fig. 6F; Fig. S2C), unaffected by Dkk1 inhibition (−22,9%, *P*=0.39). In CC, chondrocyte proliferation was increased in *Fgfr3^Y367C/+^* mutants compared to *Fgfr3^+/+^* (+188,4%, *P*<0.0001; Fig. 6G; Fig. S2D), and Dkk1 inhibition partially corrected the excessive proliferation (−39,3%, *P*<0.001; Fig. 6G).

## DISCUSSION

Genetic anomalies affecting the cartilages of the mandible can profoundly alter its development and growth, significantly impacting affected individuals with airway obstruction, feeding difficulties, sleep apnea and temporomandibular disorders (Wilkie and Morriss-Kay, 2001; Joshi et al., 2014). While mandibular growth defects caused by *FGFR3* mutations are established (Biosse Duplan et al., 2016; Morice et al., 2023), our understanding of how these mutations affect mandibular cartilage is limited, as most data come from other cartilage types like long bone growth plates, which differ in cell origin and remodeling (Parada and Chai, 2015). In our study, we specifically analyzed the transcriptome of mandibular and condyle cartilages isolated with laser microdissection. The *Fgfr3^Y367C/+^* mouse model was selected because it replicates many aspects of achondroplasia and has been used to test various therapeutic strategies (Pannier et al., 2009; Komla-Ebri et al., 2016). We concentrated on genes commonly differentially expressed in both cartilages, as chondrocyte abnormalities, particularly reduced chondrocyte differentiation, were similar in both (Biosse Duplan et al., 2016). Despite the limited tissue availability, especially for the CC, we identified several hundred differentially expressed genes in the presence of an activating *Fgfr3* mutation, including the *Dkk1* gene. The expression of Dkk1 was confirmed in cartilage sections and the *in vitro* chondrocyte model, showing nuclear, cytoplasmic, and peripheral localization. The precise localization of DKK1 in the chondrocytes of mandibular cartilage still needs to be confirmed, as well as the amount of secreted Dkk1 in the two genotypes. The overexpression of DDK1 was also observed in primary human chondrocytes from fetuses affected by thanatophoric dysplasia, another genetic disease caused by activating *FGFR3* mutations (Schibler et al., 2009). The mechanism by which *FGFR3* activating mutations lead to high DKK1 expression remains unknown. One potential pathway is through FGFR3-mediated activation of MAPK/ERK signaling, which can induce increased DKK1 expression in cancer cells (Browne et al., 2016; Niu et al., 2020). Increased MAPK signaling by the *FGFR3* activating mutation could regulate DKK1 transcription through the transcription factor CREB (Liu et al., 2015).

Dkk1 most likely plays a role in craniofacial development and is expressed in several structures, including the perichondrium of MC, the cartilage itself, and the developing mandibular bone (Nie, 2005). Its overexpression in zebrafish affects MC fusion at the midline (Alexander et al., 2014). This is could also be the case in mice, although it has not been specifically investigated (Li et al., 2006; Han et al., 2011). In humans, LRP5 gain-of-function mutations that prevent DKK1-LRP5 interaction lead to an enlarged mandible (Boyden et al., 2002).

DKK1 specifically antagonizes canonical WNT function by preventing WNT ligands from interacting with LRPs (Niehrs, 2006). In the growth plate, while necessary for the early stages of chondrocyte differentiation from mesenchymal stem cells, DKK1 inhibits hypertrophic chondrocyte differentiation (Zhong et al., 2016; Rojas et al., 2019). The chondrocytes volume enlargement to become hypertrophic determines the size of the growth plate elongation and is reduced by FGFR3 overactivation (Wang et al., 1999; Cooper et al., 2013). In mandibular cartilages, the *Fgfr3^Y367C^* mutation increases chondrocyte proliferation and reduces differentiation into hypertrophic cells (Biosse Duplan et al., 2016). In a model of mandible culture *ex vivo*, these defects were partially corrected by Dkk1 inhibition, which improved CC elongation. It should be noted that this model has limitations because it does not reproduce certain factors that influence condylar growth, such as hypoxic conditions or mechanical loading (Nickel et al., 2018). In addition, other means of DKK1 inhibition should be used to confirm the specific action of WAY262611 on DKK1. The effect of Dkk1 inhibition in *Fgfr3* mutants was more pronounced in the CC than in MC, possibly because the organization and fate of CC chondrocytes are fairly similar to those of the growth plate (Hinton et al., 2017), while most MC chondrocytes disappear at birth and do not ossify (Svandova et al., 2020). Our data confirm the different homeostasis of chondrocytes in these two cartilaginous tissues. It is striking to note that, among the differentially expressed genes between *Fgfr3* mutants and controls, only a small fraction overlapped between the two mandibular cartilages (12% of MC genes also altered in CC, and 18.5% of CC genes detected in MC), despite being sampled from the same embryos, at the same developmental stage, and in close anatomical proximity. In addition to cartilage homeostasis, it will be important to investigate whether the decreased Wnt/β-catenin signaling in *Fgfr3* mutants contributes to lower bone mass in the mandible, as well as in long bones, as previously observed (Mugniery et al., 2012). Indeed, it has been demonstrated that chondrocytes from MC produce ligands that regulate osteoblast differentiation and function (Shen et al., 2025).

Several *in vivo* studies have observed the inactivation of the Wnt pathway by FGFR3. The *Fgfr3 K650E* activating mutation in mice leads to reduced Wnt/β-catenin signaling and hypertrophic differentiation (Shung et al., 2012). In line with these findings, Fgfr3 deficiency in zebrafish upregulates canonical Wnt/β-catenin signaling, resulting in increased hypertrophic chondrocyte differentiation and craniofacial bone malformation (Sun et al., 2020). In this model, pharmacological inhibition of Wnt/β-catenin partially alleviates the phenotypes of *Fgfr3* mutants. In contrast, activating FGFRs (including FGFR3) in immortalized chondrocyte lines increased Wnt/β-catenin pathway activity, and Wnt ligands enhanced FGF-mediated suppression of chondrocyte differentiation (Buchtova et al., 2015).

Our findings suggest that Dkk1 controls chondrocyte differentiation by regulating Wnt/β-catenin activity in this ACH mouse model. Because Dkk1 also affects non-canonical Wnt signaling (Qin et al., 2024), it will be important to first specifically activate canonical Wnt signaling in the mutants using an agonist to determine whether modulation of this pathway alone is sufficient to rescue their phenotype. Targeting this pathway could be a promising pharmacological approach for improving condyle cartilage defects in conditions caused by activating *FGFR3* mutations. In this context, it will be relevant to compare inhibition of DKK1 with various therapeutic approaches tested in recent years for ACH (Legeai-Mallet and Savarirayan, 2020). DKK1 can be inhibited *in vivo* using various strategies (antibodies, miRNAs, siRNAs, oligopeptides, and small molecules) in bone diseases and cancers (Jiang et al., 2022). The DKK1 inhibitor used in this study proved effective in rescuing palatal development during embryogenesis through administration to pregnant mice (Jia et al., 2017). Whether this could be applied to craniofacial defects caused by *FGFR3* mutations would be worth exploring.

## MATERIALS AND METHODS

### Mouse models

All the experiments were conducted on *Fgfr3^+/+^* mice or *Fgfr3^Y367C/+^* littermates, as described previously (Pannier et al., 2009). Experimental animal procedures and protocols were approved by the French Animal Care and Use Committee (APAFIS#24826-2018080216094268 v5), in compliance with EU 2010/63/EU directive and complied with ARRIVE 2.0 guidelines.

### LCM

*Fgfr3^+/+^* or *Fgfr3^Y367C/+^* embryos were harvested from pregnant females at E16.5, and their heads were dissected and sectioned into two parts in the sagittal plane. Embryos showing obvious macroscopic malformations were excluded from dissection. Samples were immediately immersed in an isopentane bath at −45°C for 20 s before being placed in a sterile tube and stored at −80°C. The samples were then sectioned by cryostat along the long axis of each hemi-mandible (16 µm thickness). The sections were placed on membrane glass slides, rehydrated, stained with 0.1% Toluidine Blue and dehydrated. Using the PALM laser microdissection system (Zeiss), we visualized (20× objective) the areas of interest (MC and CC) with the staining, delineated them and harvested them on successive sections. Samples were immediately stored at −80°C. Sections that were stained but not microdissected of each embryo were also collected for RNA quality control.

### RNA-seq

Transcriptomic analysis was performed on 16 samples: eight MCs (from four *Fgfr3^+/+^* embryos and four *Fgfr3^Y367C/+^* embryos) and eight CCs (same genotype distribution). Total RNA extraction was done with an RNeasy Micro kit (Qiagen). The concentration and the purity of the total RNA extracted were measured by capillary electrophoresis using a tape station (Agilent). The strand-specific RNA-seq libraries were prepared with the Ovation Universal RNA-Seq kit (Tecan). After a preliminary DNase digestion with a thermosensitive DNase (ArcticZymes), total RNA was reverse transcribed, and second strand of cDNA was synthetized. A fragmentation step was performed before Illumina compatible indexed adaptor ligation. The ligation was followed by the strand selection enzymatic reaction to keep the information about the orientation of the transcripts. Insert Dependent Adaptor Cleavage (InDA-C) technology was used to deplete all the cDNA corresponding to ribosomal transcripts before the PCR enrichment. To ensure that no excess of amplification was performed during the final PCR step, the number of PCR cycles applied to each sample was evaluated in a preliminary Q-PCR test using EvaGreen. An equimolar pool of the final indexed RNA-seq libraries was sequenced on an Illumina HiSeq2500 (paired-end reads 130 bases+130 bases), and ∼50 million paired-end reads per library were produced.

### Bioinformatic analyses

FASTQ files were mapped to the ENSEMBL Mouse GRCm38/mm10 reference using HISAT2 and counted by featureCounts from the Subread R package. Read count normalizations and groups comparisons were performed by three independent and complementary statistical methods (Deseq2, edgeR, LimmaVoom). Flags were computed from counts normalized to the mean coverage. All normalized counts <20 were considered as background (flag=0) and ≥20 as signal (flag=1). P50 lists used for the statistical analysis regroup the genes showing flag=1 for at least half of the compared samples. The results of the three methods were filtered at *P*<0.05 or *P*<0.01 and folds 1.2/1.5/2 compared and grouped by Venn diagram. Cluster analysis was performed by hierarchical clustering using the Spearman correlation similarity measure and ward linkage algorithm.

### Primary chondrocyte cultures

Chondrocytes were isolated from MC of *Fgfr3^+/+^* and *Fgfr3^Y367C/+^* E16.5 embryos following an established protocol (Marchant et al. 2020) with modifications. The two MCs from the same embryo were dissected and digested together, first with 0.15% trypsin (Sigma-Aldrich) and 0.1% EDTA (Euromedex) in DMEM (45 min), and then by 0.15% collagenase II (Gibco) in DMEM (45 min). Finally, the cells from each embryo were resuspended in complete culture medium (DMEM/F12 with 10% FCS, antibiotics and fungizone) and plated in a well of eight-well chamber glass system (Nunc Lab Tek), with ∼7.5×10^4^ cells per well. Cells were cultured for 4 days, with the medium changed at day 2. At subconfluency (usually obtained at day 3), cells were either treated overnight with WAY2626211 (1 µM, 1 h) or appropriate controls (DMSO), or starved overnight with DMEM/F12 with antibiotics and fungizone and treated with Wnt3a (10 ng/ml or 80 ng/ml, 1 h, R&D Systems 1324-WN) or appropriate controls (BSA).

### Immunohistochemistry

Immunostaining and Hematoxylin Eosin and Safranin-O staining of cultured hemi-mandibles sections were performed as described previously (Biosse Duplan et al., 2016) with the following primary antibodies: collagen X (1/100; Diagomic 2031501005); DKK1 (1/100; Abcam 61034); PCNA (1/500; Abcam 29).

### Immunocytochemistry

Immunostaining of cultured chondrocytes was performed (Susaki et al., 2015) with the following primary antibodies: β-catenin (1/100; Invitrogen 712700); DKK1 (1/200; Invitrogen MA5-23958); Sox9 (1/500; Abcam 185230).

### Spinning disk confocal microscopy and image analysis

Images were captured using a CSU-X1 spinning disk scanner (Yokogawa) coupled to an Observer Z1 inverted microscope (Zeiss). Tile images were acquired with 20× and 40× objectives. To compare fluorescence intensity data, all confocal experiments were acquired in the same conditions. Images analysis was performed with FIJI (National Institutes of Health), by an experimenter unaware of genotypes and treatments.

### Organ cultures

Mandible cultures from *Fgfr3^+/+^* and *Fgfr3^Y367C/+^* E16.5 embryos were performed as previously reported (Biosse Duplan et al., 2016). For each embryo, the two hemi-mandibles were incubated for 6 days in DMEM with antibiotics and 0.2% BSA (Sigma-Aldrich), one supplemented with the Dkk1 inhibitor WAY262611 (Sigma-Aldrich, 317700) at the concentration of 0.5 or 1 µM diluted in DMSO (0.015 or 0.03%), whereas the other served as control and was supplemented with DMSO. Size of the mandibles and the condyles was measured at the end of time course on images captured with an Olympus PD70-IX2-UCB using CellSens software (Olympus), by an experimenter unaware of genotypes and treatments.

### Tissue clearing and immunolabeling

Cultured hemi-mandibles of *Fgfr3^+/+^* and *Fgfr3^Y367C/+^* embryos were fixed in 4% paraformaldehyde at 4°C for 24 h and decalcified in 0.5 M EDTA (pH 8.0) overnight.

Tissue clearing was achieved using the CUBIC protocol (Susaki et al., 2015). Samples were first immersed in Reagent-1 solution for 2 weeks at 37°C. After several washes with PBS, samples were blocked for 1 h at room temperature with 10% goat serum in 0.5% PBS Triton X-100. Labeling with a primary antibody (Sox9; 1/100 Abcam 185230) diluted in 0.5% PBS Triton X-100, goat serum and saponin was then performed for 7 days at 37°C. After six washes with 0.5% PBS Triton X-100 for 1 h each, a secondary antibody was used (1/100; Alexa Fluor 594, Life Technologies) overnight at 37°C. Finally, after six washes, samples were placed in Reagent-2 solution for at least 2 days and until imaging.

### Lightsheet fluorescence microscopy and image analysis

Samples were placed in 1% low-melting agarose within a capillary and images acquired with a lightsheet Z.1 microscope (Zeiss) equipped with a Plan-Apochromat 20×/NA1 R2-immersion lens objective with left and right illumination. The files obtained were first converted into an Imaris-analyzable file. After stitching the different acquisition fields of the same sample and reducing the size of the analysis field, manual segmentation of MCs and CCs was performed on successive 2D sections of the channel with Sox9 labeling using Imaris (Oxford Instruments). The volume of the segmented area was calculated, by an experimenter unaware of genotypes and treatments.

### Statistics

All values are shown as mean±s.d. Differences between experimental groups were assessed using *t*-test, or one-way ANOVA followed by, when significant (*P*<0.05), the Tukey test for multiple comparison of the mean of each group. The significance threshold was set at *P*<0.05. Statistical analyses were performed using GraphPad Prism.

## Supplementary Material

10.1242/biolopen.062540_sup1 Supplementary information
