# Supplementary material for: Dkk1 inhibition restores mandibular growth in an achondroplasia mouse model
Source: Biol Open. 2026 Apr 9;15(3):bio062540. doi: 10.1242/bio.062540 (PMC13094333; doi:10.1242/bio.062540)
Supplement: Supplementary information [file biolopen-15-062540-s1.pdf]

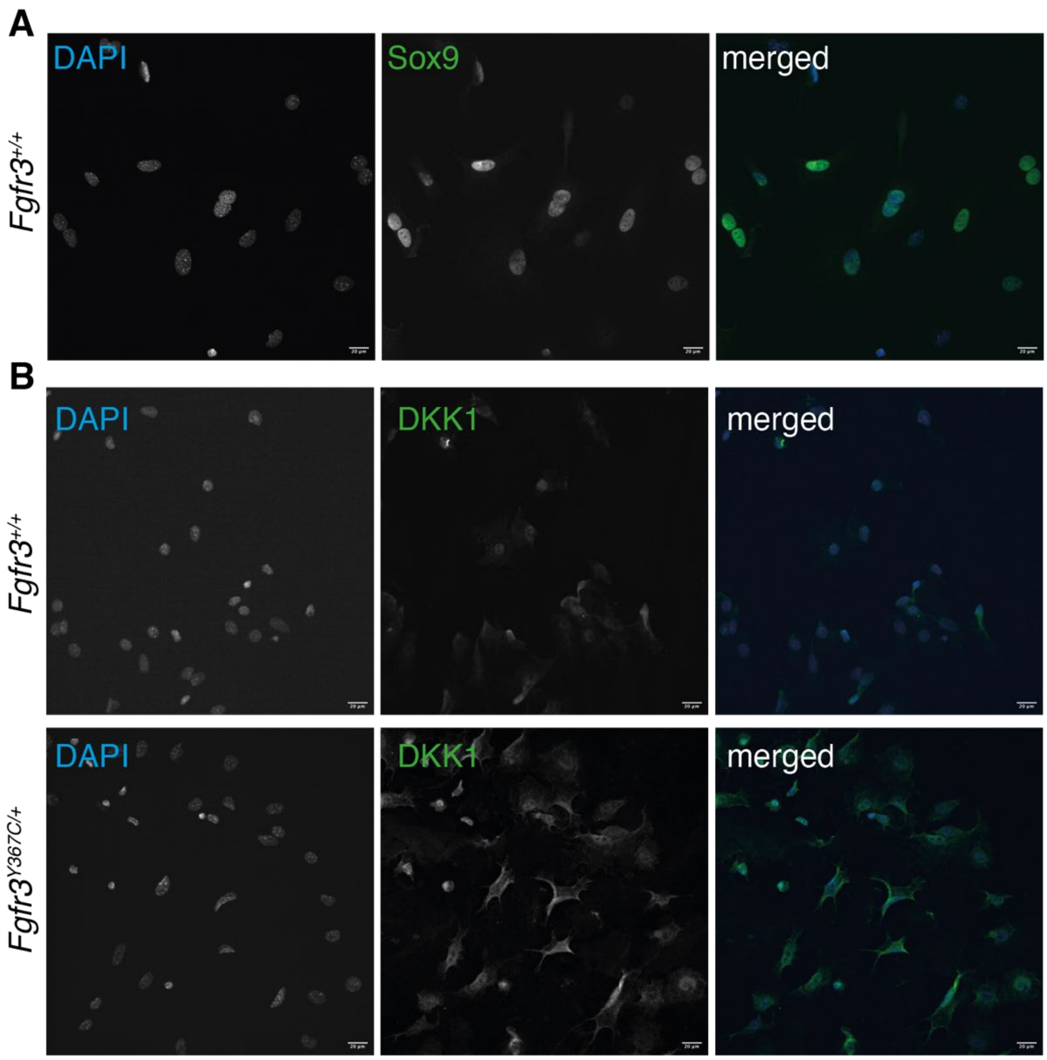

Fig. S1. Expression of Sox9 and Dkk1 in Meckel's cartilage chondrocyte cultures. (A) Visualization of Sox9 expression in cells isolated from MC of E16.5 *Fgfr3*$^{+/+}$ embryos. Scale bars are 20µm. (B) Visualization of Dkk1 expression in cells isolated from Meckel's cartilage of E16.5 *Fgfr3*$^{+/+}$ and *Fgfr3*$^{Y367C/+}$ embryos. Scale bars are 20µm.

Biology Open • Supplementary information

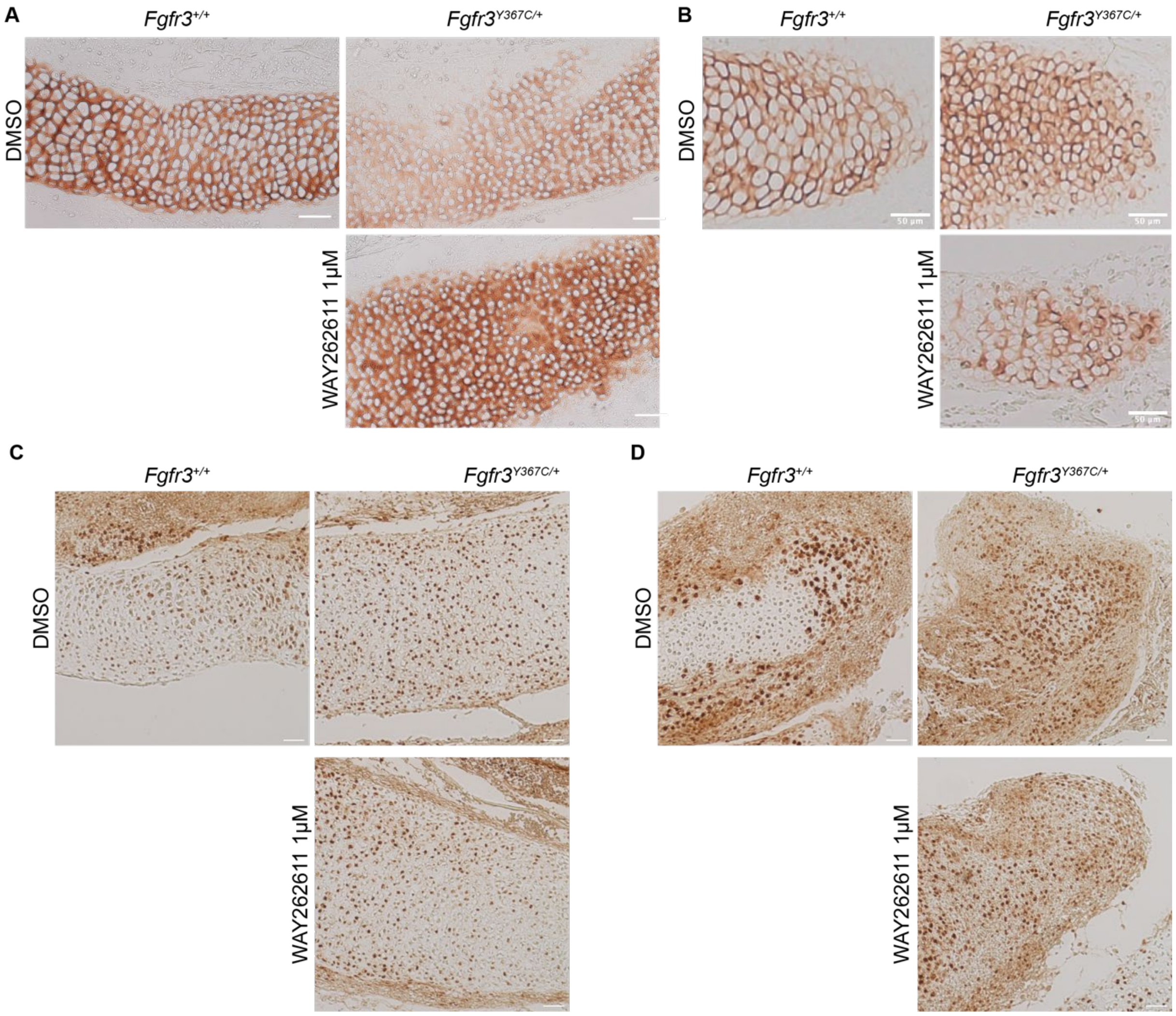

Fig. S2. Hypertrophic chondrocyte differentiation in mandibular cartilages from *Fgfr3* mutants following Dkk1 inhibition. (A) Visualization of hypertrophic chondrocytes in Meckel's cartilage of hemi-mandibles from *Fgfr3*$^{+/+}$ and *Fgfr3*$^{Y367C/+}$ embryos cultured and treated with the Dkk1 inhibitor WAY262611, following ColX immunostaining. Scale bars are 100µm. (B) Visualization of hypertrophic chondrocytes in condylar cartilage of hemi-mandibles from *Fgfr3*$^{+/+}$ and *Fgfr3*$^{Y367C/+}$ embryos cultured and treated with the Dkk1 inhibitor WAY262611, following ColX immunostaining. Scale bars are 50µm. (C) Visualization of proliferating chondrocytes in Meckel's cartilage of hemi-mandibles from *Fgfr3*$^{+/+}$ and *Fgfr3*$^{Y367C/+}$ embryos cultured and treated with the Dkk1 inhibitor WAY262611, following PCNA immunostaining. Scale bars are 50µm. (B) Visualization of proliferating chondrocytes in condylar cartilage of hemi-mandibles from *Fgfr3*$^{+/+}$ and *Fgfr3*$^{Y367C/+}$ embryos cultured and treated with the Dkk1 inhibitor WAY262611, following PCNA immunostaining. Scale bars are 50µm.

Table S1. List of genes differentially expressed in mandibular cartilages

Differentially expressed genes common in condylar cartilage and Meckel's Cartilage (n=71)	Differentially expressed genes specific of condylar cartilage (n=311)	Differentially expressed genes specific of Meckel's cartilage (n=529)
Nrgn	Ptprj	Svep1
Gm10717	Selenbp1	Arhgap28
Fap	Wnt11	Gm16351
Vnn1	Vangl1	Cntnap3
Phex	Nt5dc3	Prdm6
AB041803	Grid2	Hebp2
Hes1	Col25a1	Gtf2h5
Frem1	Gldc	Cmtm5
5430421F17Rik	Tuba1a	Angptl4
Fbln7	Wisp1	Fmn1
Igsf10	Slc35g1	Ppm1e
Nup210	Gm21807	Cachd1
Nos1	Cmbl	Bcas3
Soga2	Il1rapl2	Kcnq5
Lgi2	Slc13a5	Apex2
Trib1	Strbp	Col8a1
Tenm4	Scd2	Scin
Adamts7	Grik2	Snap91
Ppap2a	Mgat4c	Ppia
Tnfrsf11b	Csmd3	9030622O22Rik
Slc43a2	Agpat4	Spock2
Kcnq3	Rnf213	Eya1
Nhs	AK129341	Dysf
Cables1	Tgfbr3	Cd200
Gm26870	Rragd	Adamtsl3
Ttc7b	Tspan6	Col26a1
Lef1	Ift57	Ndufb9
Gm10800	Pla2g16	Igfbp7
Sfrp1	Gpr17	Adamts9
Prickle2	Esr1	Arhgap6
Slc22a23	Pla2g5	Gm12115
Fam46a	Atp1a1	Dusp6
Kctd16	Fam73b	Runx1
Col1a1	Slc16a4	Cd24a
Npr3	Glul	Rapgef5
Afap1l2	Cpeb2	Pde10a
2210408F21Rik	Dmp1	Lgalsl

Biology Open • Supplementary information

Ebf1	Gm21847	Gt(ROSA)26Sor
Gng11	Slc9a1	1810037I17Rik
Glis3	Scd1	A430005L14Rik
Thsd4	Nrg3	Ksr1
Shisa2	Znhit6	Plcxd2
Tnfrsf19	Batf3	Loxl1
Gm21738	Agbl4	Ndrg1
Gpr116	Acsf2	Dclk1
Spry2	5330426P16Rik	Slc44a5
Bmp7	Ldb2	Myo1b
Cacnb2	Lss	Kcnh1
Erg	Gls	Myo5c
Stk39	Neb	Ggh
Ston2	Kit	Trpm6
Zfp40	Gm11547	Foxn3
Aldh1a3	Gm15155	Snrpe
Pdzd2	Gpr158	Ppm1l
Gm11168	Cdh11	Ovca2
Pdlim5	Fscn1	Pitpnm3
Gm10722	Gjc3	Scara3
Dkk1	Idi1	Fam19a2
Dock9	Slc9a3r2	Maf
Gm10801	Kctd12b	Bmp3
Smoc2	Vill	Plekha7
Gm10718	Ldhb	1810041L15Rik
Spry4	Rffl	Plxdc2
Tnc	Disc1	Gm44115
Rarb	Gm4980	Adamts18
Id2	Sntb2	Spin4
Thrb	Sgms2	Egfr
Etv5	Pltp	Dusp15
Chad	Gm26827	Sparcl1
Gm17535	Nfib	Rbms3
Gm10719	Gm13318	Cidea
	Slc1a5	Kcnk10
	Rras2	Slc16a6
	Col13a1	Tbx15
	Syn1	Lamtor5
	Rasgrf2	Gm4876
	Dock3	Ppa1
	Timp1	Ttc39c
	Gm37240	Megf6

Biology Open • Supplementary information

	Tet1	Mad1l1
	Fgfr3	Col8a2
	Dcaf4	Rpl13a
	Fads3	Nrp2
	Myo5a	Dagla
	Dach1	Fam81a
	Tgfb2	9530026P05Rik
	Wnt4	Gm14634
	Lrp1b	Clmn
	Frem2	6530403H02Rik
	Arhgap18	Ostn
	Tmsb4x	2610307P16Rik
	Usp46	Epb4.1l4a
	Dmtn	Perp
	Neurl1a	Tmem261
	Gsg1l	Kctd8
	Ptpn21	Ifltd1
	Mxra8	Gm9001
	Litaf	C4b
	Sh3tc2	Fgd5
	Ldlr	Uqcrfs1
	Nrg1	Ppfibp2
	Klf12	Zbtb16
	Fam122b	Ttc18
	Stc2	Vegfc
	Ccnyl1	Mettl23
	Cacna1c	Lrrc8d
	Eogt	Tbc1d7
	Ctnna2	Acy1
	Moxd1	2810428I15Rik
	Lhfpl3	Snhg6
	Rhobtb3	Mrpl35
	Wscd2	Mrpl11
	Pax9	Ntng1
	Dync1i1	Ssbp2
	Kcns1	Rassf5
	Tbxas1	mt-Co3
	Hmgcr	C130060K24Rik
	Ebf2	Gm11263
	Klhl30	C4a
	Loxl3	Mir351
	Prom1	Jag1

Biology Open • Supplementary information

	Celf2	Ust
	Lfng	Nebl
	Spock1	Rpl35a-ps4
	Ptprz1	Slit2
	Rara	Pou3f2
	Pcdh18	Col6a2
	Enpp6	Pde7b
	Map2	Neat1
	Tmem40	Gem
	Abhd12	Tmem121
	Dmd	Fcer2a
	Slc45a4	Fbxl7
	Capn1	Pin4
	Orai1	Ntn1
	Dhx32	Tspan5
	Katnal1	Atp6v0a4
	Ttc7	Lgr6
	Tacc2	Magi1
	Gm20559	Dock5
	Cds1	Ndufb4
	Tbc1d16	Btg2
	Grin2b	Enpep
	Fasn	Myo10
	March1	Arhgap42
	Loxl2	Gpn1
	Serpinf1	Etv4
	Bmpr1b	Gm15655
	Insig1	Setbp1
	Fzd9	Trim59
	Ntrk3	Plce1
	Rapgefl1	Cp
	Acpl2	Zeb2
	Mgll	Adam12
	Cntnap2	Cryl1
	Heatr2	Wdr96
	A530021J07Rik	Pard3
	Pros1	Lsmd1
	Crip2	Ibsp
	Slc7a11	Cd163l1
	Atp8a2	Srgap3
	Orai2	Esco2
	Adrbk2	Loxl4

Biology Open • Supplementary information

	Pik3ip1	Cib2
	Scarb2	Prdx4
	Papss2	Sema3e
	Cln6	Tmem132b
	Frk	Ogn
	Hsd17b7	Dock4
	Rab2b	Ptpn13
	Scn1b	Nhp2l1
	Ccrn4l	Slco2a1
	Sybu	Rpl15
	Mcm10	Papln
	Cadm2	Calcrl
	Thbs3	Eda2r
	Ccdc88c	Fam134b
	Nav2	Fgd6
	Unc5c	Rpl39
	Ctsk	Fosl2
	Fam65b	Plcl1
	F5	Robo1
	Acan	Sulf1
	Tssc1	Rpl7a
	Atrnl1	Gm14582
	Bmp6	Ptpru
	Lpl	Cmip
	Nrxn1	Pvt1
	Fxyd1	Commd3
	Hmga2	Col6a1
	Chrm3	Kif26a
	Ctnnd2	Col14a1
	Foxo4	Gm9493
	Opcml	Prorsd1
	Adarb1	Lamb1
	Pde4b	Rpa3
	Maml2	Steap1
	Kcnk1	Tspan8
	Phyh	Arvcf
	Rbms1	Angpt1
	Il6ra	5730522E02Rik
	Extl1	Tjp2
	Synpo2	Adcy5
	Spp1	Baiap2l2
	Stk38l	Gm8430

Biology Open • Supplementary information

	Proser2	Ttll3
	Hip1r	Prr5l
	Phactr1	Igfbp5
	Mest	Ttc12
	Pygl	Arl15
	Mvd	Tmem150c
	Lingo2	Gria1
	Itpr2	Tshz2
	Sema3a	Zeb1
	Kctd5	Afap1
	Dcbld2	Cadm1
	Fam171b	Mkl1
	Enpp1	Sall1
	Klf15	Nbl1
	Tiam2	Htra4
	Plac1	Cdh6
	Fcgrt	Foxa3
	Tmem163	2010107E04Rik
	Prss12	Lpar1
	Chsy1	Nutf2-ps1
	Slc14a1	Uqcrb
	Ifngr2	Tnfrsf1b
	Mgp	Mycbp
	Cytl1	Pik3c2b
	Rasa3	Sfrp2
	Nsdhl	9930013L23Rik
	Me1	A2m
	Vipr2	Zfp521
	Srpx2	Ccne2
	Itga8	Arap2
	Fam102b	Col12a1
	Agtrap	Nos1ap
	Fam89a	Gm22513
	Notch1	Dlx1
	Gpm6b	Mrc1
	Abca9	Agap2
	Kcnh7	Atf3
	Osbpl6	Skap2
	4931406P16Rik	Rpl36a-ps1
	Adarb2	Errfi1
	Zfp804a	Lemd1
	Fign	Mrpl16

Biology Open • Supplementary information

	Shisa4	Gas1
	Serinc5	Rab3il1
	Pcdh15	Mocos
	Igsf3	Lamc3
	Cadps2	Arhgap24
	Pxdc1	Csrp2
	Relt	Igfbp4
	Dmpk	Ptprv
	Lama2	Sema3d
	Prkcdbp	RP23-103I12.3
	Stard10	S1pr1
	Epdr1	Gm12811
	Nfasc	Glis1
	Lpin1	Itga4
	Robo2	Rps27
	Nespos	Avpr1a
	Mbp	Cgnl1
	Dmwd	Spry1
	Lipg	A230065H16Rik
	Car8	Klf5
	Rgcc	Sft2d3
	A330008L17Rik	Miat
	Ak4	Ldlrad4
	Tnfsf13b	Zfp503
	Thra	Gm10721
	Foxp2	Fam49a
	Net1	Ptprm
	Ppfia2	Sec11c
	Gfpt2	Psmc3ip
	Grasp	Clu
	Gcnt2	Pced1b
	Asap2	Tll1
	Dhcr24	Sh2d4a
	Bdnf	Amd2
	Lrrk2	Dcaf12l2
	Dpysl3	Acsm3
	Pim1	Dusp1
	Bri3bp	Exoc3l4
	Enc1	Pik3cb
	Gdf10	Sorcs2
	Enpp2	Slit3
	Pmepa1	Smoc1

Biology Open • Supplementary information

	Phf15	Rai14
	Fryl	Atp5h
	Abca8a	Fam20a
	Fads2	Gdf5
	Ntm	D930015E06Rik
	Slc6a9	Cilp2
	Slc29a1	Ccdc162
	Slc43a3	Ppfia4
	Tln2	Foxa2
	Ankrd6	Matn3
	Prdm16	Txnl4b
	Nav1	Gm4294
	A930015D03Rik	Siva1
	Trerf1	Rab11fip5
	Alox12	D10Bwg1379e
	Rin2	Flrt2
	Appl2	Cd55
	Ndrg4	Lyrm2
	Cdh18	Pdp1
	Pde11a	Ndufa4
	Acly	Gm24305
	Fgf14	Mgmt
		Ephb2
		Dkk2
		Apbb2
		Slc24a3
		Celsr1
		Smpdl3a
		Chst2
		Adam19
		Mical2
		Gm26858
		Gm19744
		Alcam
		Csrnp3
		Il1rapl1
		Rnf180
		Htra1
		Ube2e2
		Hmcn1
		Scn9a
		Nckap5

Biology Open • Supplementary information

		Snx29
		Rcan2
		4632428N05Rik
		Mis18a
		Gm13052
		Arxes2
		Gm10720
		Mitf
		Tmem47
		Erh
		Aldh1a2
		Otor
		Naa38
		Bicc1
		Tmub1
		Gm44280
		2900026A02Rik
		Bmp5
		Rnase4
		Cyba
		Gm22317
		Pbk
		March3
		A4galt
		Tenm3
		Scara5
		Lum
		Nop10
		Tmem126a
		Trp53i11
		Mypn
		Camta1
		Cacybp
		D630045J12Rik
		Gm10715
		Gm7120
		Usmg5
		Fndc1
		Ninj2
		Rpl22l1
		St8sia2
		Snhg12

Biology Open • Supplementary information

		Mylk
		Atg10
		Rpl30-ps8
		Fgf12
		Aldoc
		Akap12
		Ablim1
		Gm14295
		Mrpl32
		2810001G20Rik
		Cdh2
		Cubn
		Gm12942
		Svopl
		2810055G20Rik
		Pde4d
		Polr2d
		Gm24130
		Tspan12
		Tmem132c
		Fam167a
		Adcy4
		Rps27l
		Cetn2
		Hrc
		Lsm7
		Lama5
		Socs2
		Trmt10c
		Rps27a-ps2
		Fam72a
		Fras1
		Arxes1
		Ddt
		Col6a6
		Bex2
		Pard6g
		Serpine1
		BC003965
		Mtss1
		Npas2
		Kcnt2

Biology Open • Supplementary information

		Tmem107
		KCTD12
		Rerg
		Iffo2
		Cmklr1
		Sgip1
		Tmem42
		Ifitm10
		Lama1
		Lypd6
		Etl4
		Exoc4
		Ptgfr
		Ndufs5
		Nxt2
		Cacna1g
		Lhfp
		Ttc30a1
		Itga11
		Ppp1r3c
		Gm13507
		Sctr
		Mecom
		Gm29266
		Itga1
		Fhl2
		Pkp4
		Klhdc8a
		Flt4
		Mmp9
		Shank2
		Adamtsl4
		Iqgap2
		AV026068
		Gab2
		Snrpg
		Zswim6
		Cenpp
		Hivep2
		Meis2
		Esrrg
		Mrps14

Biology Open • Supplementary information

		Tomm5
		B830012L14Rik
		Kirrel3
		Kcnq4
		Slc16a9
		Ptprd
		Creb5
		Rpl35a-ps3
		Pard3b
		Sox13
		Sytl5
		Kazn
		Gm10177
		Nrtn
		Slc25a30
		Abi3bp
		Rftn1
		Pde9a
		Ltbp2
		Aox1
		Aff3
		Tnik
		F630206G17Rik
		Pamr1
		Tmcc3
		Ucp2
		Galnt15
		Prg4
		Mllt3
		Rpl36a
		Gm9846
		Ccrl2
		Pcsk2
		Runx1t1
		Elfn1
		Rbpms
		Vdac3-ps1
		Nlgn3
		Trabd2b
		Rbfox3
		Rab30
		Wnk2

Biology Open • Supplementary information

		Lcp1
		Cthrc1
		B3galt1
		Gm4895
		Rps6ka5
		Gm7628
		Samd12
		Anxa3
		Polr2k
		Rps27a
		Sgpp1
		Tspan11
		Cenpq
		Fmnl2
		Gm14253
		Fam227b
		AI661453
		N6amt1
		Cav2
		Igf1
		Lrrc8b
		Zdhhc14
		Gm9790
		Raf1
		Svil
		Dr1
		Dnaaf2
		H3f3a
		Penk
		Efna5

List of genes differentially expressed in mandibular cartilages between *Fgfr3*$^{+/+}$ and *Fgfr3*$^{Y367C/+}$ embryos (filters: P<0.01, mean expression ratio >2). The first column lists the genes that are differentially expressed in both Meckel’s and condylar cartilages, the second column lists genes differentially expressed in condylar cartilages only and the third column the genes differentially expressed in Meckel’s cartilages only.

Biology Open • Supplementary information
